# Pronouncing Medical Lexicon

**Published:** 1856-09

**Authors:** 


*Pronouncing Medical Lexicon.* By C. H. Cleaveland, M. D., Cincinnati.

We have received a second copy of this apology for a dictionary, from which we regret to infer that it has reached a second edition. Our unfavorable opinion of its merits has been already expressed, and it is unnecessary to repeat it.

